# *Salmonella* Enteritidis ST183: emerging and endemic biotypes affecting western European hedgehogs (*Erinaceus europaeus*) and people in Great Britain

**DOI:** 10.1038/s41598-017-18667-2

**Published:** 2018-02-05

**Authors:** Becki Lawson, Lydia H. V. Franklinos, Julia Rodriguez-Ramos Fernandez, Clare Wend-Hansen, Satheesh Nair, Shaheed K. Macgregor, Shinto K. John, Romain Pizzi, Alejandro Núñez, Philip M. Ashton, Andrew A. Cunningham, Elizabeth M. de Pinna

**Affiliations:** 1 Institute of Zoology, Zoological Society of London, Regent’s Park, London, NW1 4RY United Kingdom; 2 Gastrointestinal Bacteria Reference Unit, Public Health England, London, NW9 5EQ United Kingdom; 3 Scottish SPCA National Wildlife Rescue Centre, Fishcross, Alloa, FK10 3AN, & Royal Zoological Society of Scotland, Edinburgh, EH12 6TS United Kingdom; 4 Animal and Plant Health Agency (APHA), APHA Weybridge, New Haw, Addlestone, Surrey, KT15 3NB United Kingdom; 5 Present Address: University College London, Gower Street, London, WC1E 6BT UK; 6 Present Address: IDEXX Laboratories Limited, Grange House, Sandbeck Way, Wetherby, West Yorkshire, LS22 7DN UK

## Abstract

The impacts of hedgehog (*Erinaceus europaeus*) *Salmonella* infection on public health and on animal welfare and conservation are unknown. We isolated *Salmonella* Enteritidis multi-locus sequence-type (ST)183 from 46/170 (27%) hedgehog carcasses (27 *S*. Enteritidis phage type (PT)11, 18 of a novel PT66 biotype and one with co-infection of these PTs) and from 6/208 (3%) hedgehog faecal samples (4 PT11, 2 PT66) from across Great Britain, 2012–2015. Whole genome phylogenetic analysis of the hedgehog isolates and ST183 from people in England and Wales found that PT11 and PT66 form two divergent clades. Hedgehog and human isolates were interspersed throughout the phylogeny indicating that infections in both species originate from a common population. PT11 was recovered from hedgehogs across England and Scotland, consistent with endemic infection. PT66 was isolated from Scotland only, possibly indicating a recent emergence event. People infected with ST183 were four times more likely to be aged 0–4 years than people infected by the more common ST11 *S*. Enteritidis. Evidence for human ST183 infection being non-foodborne included stronger correlation between geographic and genetic distance, and significantly increased likelihood of infection in rural areas, than for ST11. These results are consistent with hedgehogs acting as a source of zoonotic infection.

## Introduction

*Salmonella enterica* serovar Enteritidis (*S*. Enteritidis) is one of the commonest types of *Salmonella* to infect people and animals^1,2^. The majority of human *S*. Enteritidis infections in England and Wales are due to sequence type (ST)11, but ST183 accounted for circa 3% of infections, April 2014-December 2015 (Public Health England (PHE) data, provisional). Contaminated food is the most common source of human *Salmonella* infection in Great Britain (GB)^3^, however there are examples of zoonotic infection from both captive and free-living wildlife; for example *Salmonella enterica* subspecies *arizonae* from pet reptiles^4^ and *Salmonella* Typhimurium from wild birds^5,6^.

The western European hedgehog (*Erinaceus europaeus*) is a small nocturnal insectivore whose GB population has declined over recent decades^7^. The reasons for this decline are incompletely understood but believed to be multi-factorial, including habitat fragmentation, food shortage, road traffic collision injuries and predation. Whether infectious or non-infectious disease is a contributory factor is unknown. Hedgehogs are the most frequently admitted mammal to wildlife rehabilitation centres in GB^8^ and supplementary feeding of hedgehogs in gardens is a popular activity. Opportunity therefore exists for close contact between hedgehogs and people, with potential for zoonotic and anthroponotic pathogen transmission^9–11^.

*Salmonella* infection has been described in free-living hedgehogs from GB and continental Europe, including with *S*. Enteritidis phage type (PT)9a and PT11 and with *S*. Typhimurium definitive type 104^11–14^. These studies typically describe small numbers of affected animals, are limited to a particular geographical region, and/or focus on casualty animals in care at wildlife rehabilitation centres^12,15^. *Salmonella* Enteritidis PT11 infection has been most frequently reported and has been proposed as being endemic in the hedgehog^12^. There have been no recent studies exploring *Salmonella* biotypes in free-living hedgehogs in GB, and there is a paucity of data to inform the wildlife and public health implications of these infections. Elsewhere, infection of both hedgehogs and people with *S*. Enteritidis PT11 and PT9a in Denmark and *S*. Typhimurium in Norway^13,14^ has been found, with the authors postulating that the hedgehog acts as a primary source of human infection in these countries for these biotypes.

Here we screened a convenience sample of hedgehog tissues and faecal samples, collected from across GB, 2012–2015, for *Salmonella* spp. infection. We used whole genome sequencing (WGS) of all *Salmonella* spp. isolates and compared them with human-derived isolates of matched biotypes to investigate phylogenetic relationships and evidence for spatial structuring. Lesions associated with *Salmonella* infection in hedgehogs are summarised and the results of antibiotic sensitivity testing on isolates from hedgehog and humans are presented. Finally, we also explore the demography of human infections with these matched biotypes and use this to appraise public health implications.

## Methods

### Hedgehog samples for *Salmonella* testing

#### National Scanning Surveillance Programme

Opportunistic reports of hedgehog mortality were solicited from members of the public from across Great Britain, August 2012 - December 2015 inclusive, as part of a national scanning surveillance programme. All available carcasses, regardless of suspected cause of death, were examined. Carcasses were refrigerated at 4 °C on arrival at the Institute of Zoology (IoZ) and examined fresh where possible; they were otherwise archived at −20 °C for later examination. Post-mortem examinations (PMEs) were conducted following a standardised protocol, with systematic external and internal examination of all body systems. Sex, age and body weight were recorded.

The liver, small intestinal contents and any macroscopic lesions were routinely cultured for pathogenic bacteria, including for *Salmonella* spp. using *Salmonella*-selective enrichment media^6^ (see Supplementary Methods S1). Bacterial isolates were identified using colonial and Gram staining morphology coupled with biochemical characteristics determined using the analytical profile index (API) 20 Enterobacteriaceae biochemical test strip method (API-BioMerieux, Marcy l’Etoile, France). Slide agglutination tests were performed for the identification of suspected *Salmonella* spp. isolates using poly-O antisera (Pro-lab diagnostics, Neston, UK). *Salmonella* isolates, each grown from a single colony, were placed onto microbank beads (Pro-lab diagnostics) and stored at −80 °C.

A full suite of tissues and lesions were fixed in 10% neutral buffered formalin and prepared for histopathological examination using standard techniques, where permitted by the state of tissue preservation. Tissue sections were stained, using routine methods, with Haematoxylin and Eosin. Selected sections were stained with Gram, Periodic Acid-Schiff and/or Ziehl-Neelsen stains as indicated on examination. Also, immunohistochemical demonstration of *Salmonella* common structural antigens (CSA-1) was conducted on a subset of cases (see Supplementary Methods S2).

#### Wildlife centre casualty admissions based on syndromic surveillance

The Scottish Society for the Protection of Animals National Wildlife Rescue Centre (NWRC) in southern Scotland first detected an apparently novel clinical presentation of hedgehog casualties in 2008. Hedgehogs with large palpable abdominal masses, suspected to be enlarged and/or abscessated mesenteric lymph nodes, were identified and euthanased on welfare grounds. Affected carcasses obtained in 2012–2014 were stored frozen prior to PME following the above protocol. In 2015, similarly-affected fresh carcasses were examined post mortem when tissues suitable for histopathological examination were obtained.

Macroscopic and microbiology findings were reviewed for each hedgehog, in combination with histopathology results when available. Hedgehogs without histopathological examination were considered as likely having salmonellosis (i.e. infection causing disease) when macroscopic evidence of enlarged and/or abscessated lymph nodes or enteritis was detected with *Salmonella* sp. recovered from the affected sites or organs. When *Salmonella* sp. was isolated in the absence of macroscopic abnormalities, the significance of the infection remained unknown if histopathological examination was not performed. If suppurative, granulomatous and/or necrotising inflammation was detected microscopically in at least one of the tissues from which *Salmonella* sp. was isolated, a diagnosis of salmonellosis was made; if these changes were not observed, the significance of the infection remained unknown.

#### Faecal samples

A network of eight wildlife casualty treatment and rehabilitation centres located across GB was recruited for the collection of hedgehog faecal samples^16^. During the periods April-June 2014 and June-September 2015, a single, freshly-voided faecal sample was collected from each hedgehog casualty soon after admission (most within 48 hours). The date and approximate location of origin were recorded for each hedgehog. In addition, faecal consistency, colour and the presence/absence of blood were described by the submitter. Samples (minimum 1 g) were stored at 4 °C for up to one week before submission to IoZ for bacteriological examination, as above.

### Selection of *Salmonella* from human samples

All isolates from humans received by PHE between July 2012 and March 2016 characterised as ST183 were included in the study. As part of a separate validation project^17^, a structured sample of 1500 *Salmonella* received by PHE between April 2012-March 2013 were sequenced, the nine ST183 from this sample were included in this study. From April 2014 onwards, all *Salmonella* received by PHE were sequenced of which 140 were identified as ST183. Along with date of receipt by PHE (used as a proxy of the date of isolation), patient age, geographical region and home post code were extracted where available. Whilst access to human patient histories was not possible as part of this study, the vast majority of human ST183 infections were isolated from faecal samples which implies the presenting symptoms were gastrointestinal.

### Characterisation of *Salmonella* sp. isolates

Batches of *Salmonella* spp. isolates were submitted to the *Salmonella* Reference Service (SRS) of PHE where standardised international protocols were used for biotyping (serotyping^18^ and phage typing^19^). Antibiotic sensitivity testing was performed at the SRS using a breakpoint method^20^. The antibiotics tested are listed in Supplementary Methods S3.

Cultures were grown, DNA extracted, whole genome sequencing (WGS) performed and data quality controlled for all *Salmonella* sp. isolates (see Supplementary Methods S4). The multi-locus sequence type (ST) was determined from FASTQ files using the MOST v1.0 software package which was used to infer the serotype^17,21,22^.

### Phylogenetic and statistical analyses

WGS single nucleotide polymorphism (SNP) phylogenetic analysis was performed on the hedgehog *Salmonella* spp. isolates and from human ST183 isolates for which WGS data were available. Briefly, phylogenies were determined by mapping the short reads against the *S*. Enteritidis P125109 reference genome^23^ (NCBI accession AM933172) using BWA mem v0.7.12^24^; SNPs were then called using GATK v2.6.5^25^ in unified genotyper mode. A consensus genome was called for each isolate, with positions with <10 reads mapped or an Mapping Quality of <30 defined as Ns. SNPs at positions that were present (i.e. not N) in 80% of isolates and which passed quality criteria of >90% consensus, minimum depth 10x, GQ > = 30 and MQ > = 30 in at least one strain were extracted and used to derive a consensus genome for each isolate. Regions of the reference genome corresponding to pro-phage regions, as identified by PHAST^26^, were masked. These regions were 920105-949171, 1013332-1021872, 1222614-1257489, 1453431-1492466, 2007699-2072028 of the AM933172.1 reference. This whole genome alignment was then used as input for Gubbins to remove potential recombinant regions^27^ and the Gubbins filtered polymorphic positions used to generate a maximum likelihood phylogeny with IQ-TREE v1.3.10^28^. Phylogenies were visualised with phandago^29^. Raw FASTQ data was uploaded to the NCBI SRA BioProject PRJNA248792, with sample specific accessions available in Supplementary Table S1.

The relationship between spatial distance and genetic distance for ST183 was calculated in the following way (after Figure 7 of^30^). A SNP pairwise distance matrix was produced for all available ST183 from humans. Then a pairwise distance matrix consisting of straight line geographic distance in kilometres was produced for all human isolates with an associated postcode using the postal area (first half of postcode) translated into an XY co-ordinate and the distance between them calculated according to Pythagoras’ theorem. Then, for a threshold of each SNP distance in the range 0-100, the number of pairs with a SNP distance less than or equal to the SNP threshold that were also within 30 km of each other in the geographic distance was expressed as a proportion of the total number of pairs with SNP distance less than or equal to the threshold. 100 random permutations of the same data were used to show signal over noise. In order to address the hypothesis that ST183 had a stronger geographical relationship than ST11, a control set of 71 ST11 isolates was analysed. It was not feasible to carry out the permutation analysis with the entire collection of ST11 in the PHE database, as this consisted of thousands of isolates. The control set was selected on the following criteria (1) similar pairwise SNP distance distribution to that of all ST11 (Supplementary Figure S1) and (2) similar maximum pairwise SNP distance as within ST183.

The demography of human infections with PT11 and all non-PT11 *S*. Enteritidis infections was summarised. The home address of each human ST183 isolate and 2060 ST11 isolates from Jan 1^st^ to December 31st 2015 were classified as rural or urban based on the patient post code using ArcGIS v10.

Antimicrobial resistance determinants were sought using ‘Genefinder’, a PHE program that uses Bowtie 2^31^ to map reads to a set of reference sequences representing typically acquired antibiotic resistance genes and chromosomal regions involved in antibiotic resistance and SAMtools to generate an mpileup file. The mapping data were parsed, and reference sequences with 100% coverage, >85% base-call variation and >90% nucleotide identity were called as present in the genome, while allowing for detection of novel sub-types. β-lactamase variants were determined with 100% identity using the reference sequences downloaded from the Lahey (www.lahey.org) or NCBI β-lactamase data resources (https://www.ncbi.nlm.nih.gov/pathogens/beta-lactamase-data-resources). Known acquired resistance genes and resistance-conferring mutations relevant to β-lactams (including carbapenems), fluoroquinolones, aminoglycosides, chloramphenicol, macrolides, sulphonamides, tetracyclines, trimethoprim, rifamycins and fosfomycin were included in the analysis^32^.

### Retrospective data review

The Scottish *Salmonella* reference laboratory (Stobhill) and the Animal Plant & Health Agency (APHA), who conduct *Salmonella* biotyping of human and non-human isolates, were contacted to determine if they had isolated a variant PT15 isolate (subsequently identified as a novel PT66 from hedgehogs in this study) and, if so, from which host(s). In addition, SRS databases (2004–2015) and APHA databases (2002–2016) were reviewed to identify non-human species from which the *Salmonella* sp. phage types recovered from hedgehogs have been isolated in England and Wales.

## Results

### Hedgehog *Salmonella* infections: pathological findings and faecal microbiology results

PMEs were conducted on 170 hedgehogs from across GB, August 2012-December 2015 inclusive: 151 from the scanning surveillance programme and 19 from the NWRC. The only *Salmonella* spp. identified was *S*. Enteritidis, which was isolated from 46 hedgehogs: 26 from the scanning surveillance program and 20 from the NWRC (see Supplementary Tables S1 and S2). No other significant bacteria were isolated. Two *S*. Enteritidis phage types were identified: PT11 (n = 27, all but one from the scanning surveillance programme) and PT66 (n = 18, all from the NWRC in Scotland). One hedgehog from the NWRC had concurrent infection with both phage types. The sex, age and seasonal distribution of hedgehogs with *Salmonella* infection are summarised in Supplementary Table S3. Originally PT66 was thought to be a variant of PT15. The PT11 and PT66 phage types only differ in their reaction with one phage. However WGS determined PT15 isolates to be ST11 and PT66 isolates to be ST183. Consequently, PT66 was assigned as a new phage type.

The macroscopic abnormalities of each hedgehog with *Salmonella* infection are summarised in Supplementary Tables S1 and S2. Briefly, a variety of macroscopic abnormalities were observed in hedgehogs with PT11. Abnormalities in the seven hedgehogs with PT11 confirmed with salmonellosis comprised mesenteric lymph node (MLN) enlargement (5/7), MLN abscessation (5/7), gastritis (4/7), enteritis (2/7), peritonitis (1/7) and pyelonephritis (1/7). Additionally, abnormalities were found that are not typically or specifically associated with *Salmonella* sp. infection, such as traumatic injuries (e.g. haemorrhage and fractures) n = 4, parasitic pneumonia n = 8, periodontal disease n = 2 and heavy tick burden n = 4.

**Figure 1:**
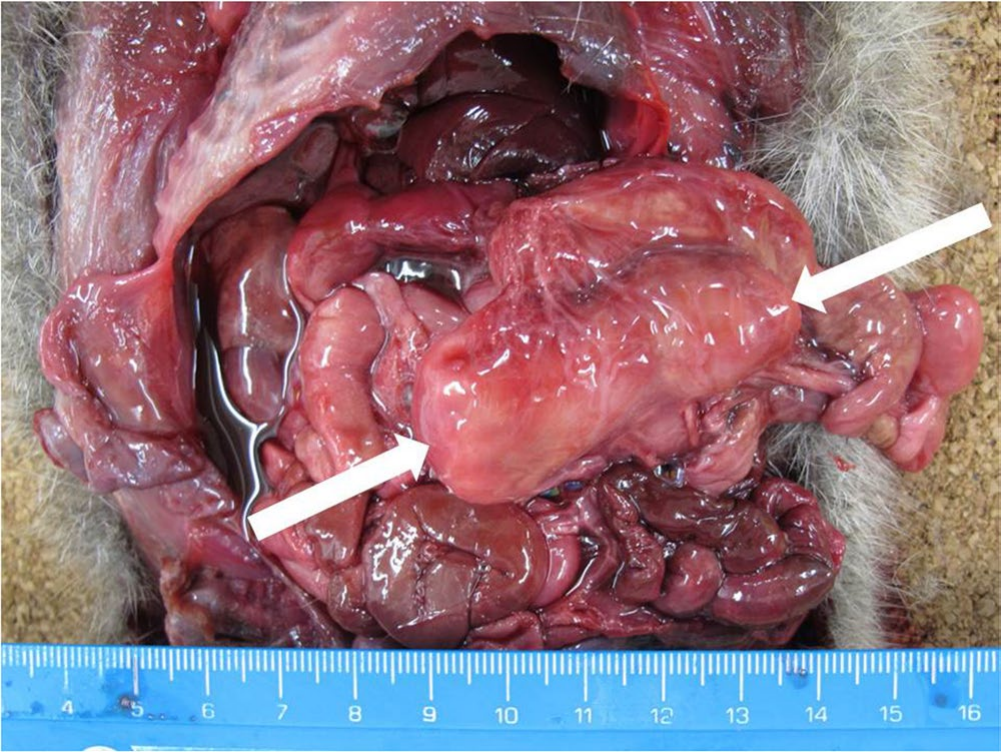
Enlarged mesenteric lymph node in a hedgehog with *Salmonella* Enteritidis PT66 infection. White arrows denote the maximum dimensions (circa 6 cm long).

All the PT66 hedgehogs were submitted from the NWRC and, therefore, according to the case selection criteria, all had MLN enlargement (Fig. 1) with a mean maximum MLN dimension of 52 mm (range 21–83; n = 19) (compared to 37 mm (range 8–60; n = 10) for PT11 cases). All but one of the PT66-infected animals had gross MLN abscessation. Gross evidence of enteritis was noted in one hedgehog and of gastritis in another. Abscesses in other sites were present in eight of the PT66 hedgehogs: these comprised adrenal gland (1/8), axillary LN (1/8), mediastinal LN (4/8), submandibular LN (1/8) and thymus (1/8). Granulomatous pneumonia was noted in three hedgehogs; PT66 was recovered from the lung of two of these animals.

**Figure 2:**
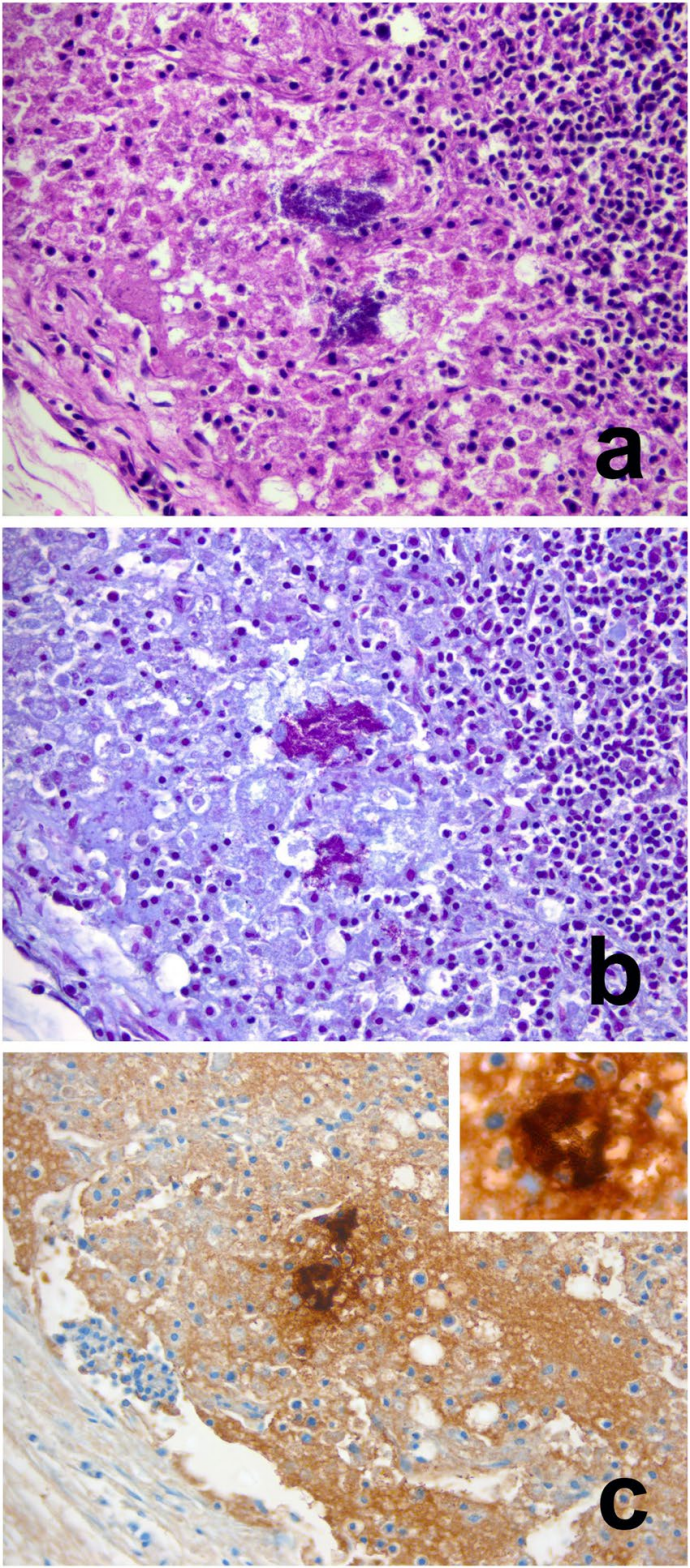
Histopathology and immunohistochemistry of hedgehog with *Salmonella* Enteritidis PT66 infection. (**a**–**c**) Serial sections of mesenteric lymph node from hedgehog XT-1053-15. (**a**) Necrotizing lymphadenitis in subcapsular areas with abundant intralesional bacterial colonies. Haematoxylin and Eosin. 400x. (**b**) The bacteria are Gram negative. Gram Twort. 400x. (**c**) The bacteria show immunoreactivity for *Salmonella* CSA-1. Inset: detail of bacterial immunolabelling. IHC. Ventana. 400x.

Microscopic findings from the PMEs are summarised in Supplementary Tables S4 and S5. Briefly, hedgehogs with PT11 (n = 8) presented a wide variety of microscopic lesions, including verminous bronchitis and bronchiolitis, verminous tracheitis, myositis, nephritis and cystitis. Lymphadenitis with intralesional bacteria (Fig. 2) was present in all three hedgehogs with PT66 examined histologically. The tissues of the hedgehog with PT11 & PT66 co-infection were too autolysed for meaningful histopathological examination. The IHC demonstrated specific immunolabelling of intralesional bacteria (Fig. 2c) in multiple tissues (MLN, retropharyngeal lymph node, adrenal gland, thymus and lung) with inflammatory lesions from three animals: 2 with PT66, 1 with PT11 (see Supplementary Tables S4 and S5).

**Figure 3:**
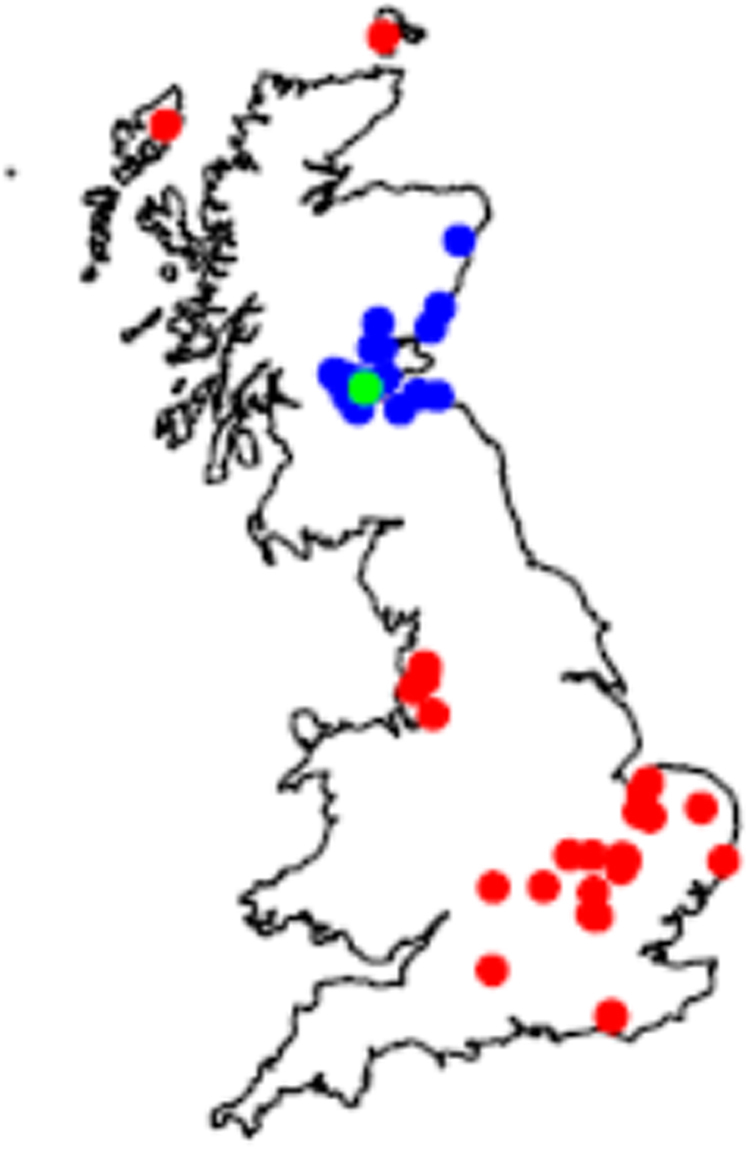
Distribution of ST183 hedgehog infections (post-mortem examination and faecal samples combined) from Great Britain, 2012-2015. Red circle PT11, blue circle PT66, green circle co-infection with PT11 & PT66. Map was generated using R version 3.3.2 (2016-10-31) available to download at https://cran.r-project.org/bin/windows/base/. R core team. *R: A language and environment for statistical computing*. (R Foundation for Statistical Computing, 2015).

These results led to a diagnosis of salmonellosis in 26% (7/27) of PT11 hedgehogs and all 19 PT66 hedgehogs. The significance of the *Salmonella* infection remained undetermined in the remaining PT11 cases. Hedgehogs with *S*. Enteritidis PT11 infection had a widespread distribution across GB whilst PT66 was only recovered from cases in southern and central Scotland (Fig. 3). *Salmonella* Enteritidis isolates with a variant PT15 were submitted from Stobhill and APHA and subjected to WGS: this confirmed the one isolate from a hedgehog in England was in fact PT11 and the three human isolates and one hedgehog isolate from Scotland were PT66.

A total of 208 hedgehog faecal samples were screened (131 in 2014, 92 in 2015) with a wide spatial distribution across GB, of which 7 (3%) were positive for *Salmonella* sp. infection (4 PT11, 2 PT66 and a single *Salmonella* Agama) (Fig. 3). Both PT66 isolates were from eastern Scotland, the PT11 isolates were from the East of England (n = 3) and south east Scotland (n = 1) and the *S*. Agama isolate was from the East of England. A description of the faecal appearance was available for five of the culture-positive samples, four of which were considered abnormal (runny and/or mucoid and/or green colour).

### Phage typing and whole genome SNP phylogeny

Sequences from 149 ST183 isolates from sporadic human infections, obtained between July 2012 and March 2016, were available from PHE. Additionally, 47 isolates from hedgehogs (41 from PMEs and six from faeces samples in this study) were sequenced by PHE. Phage typing information was available for 186 of these 196 isolates, providing six different phage types (see Supplementary Worksheet S1). The most common PTs were PT11 (n = 141) and PT66 (n = 28), with one mixed PT11 and PT66 infection from which the PT11 isolate was sequenced.

**Figure 4:**
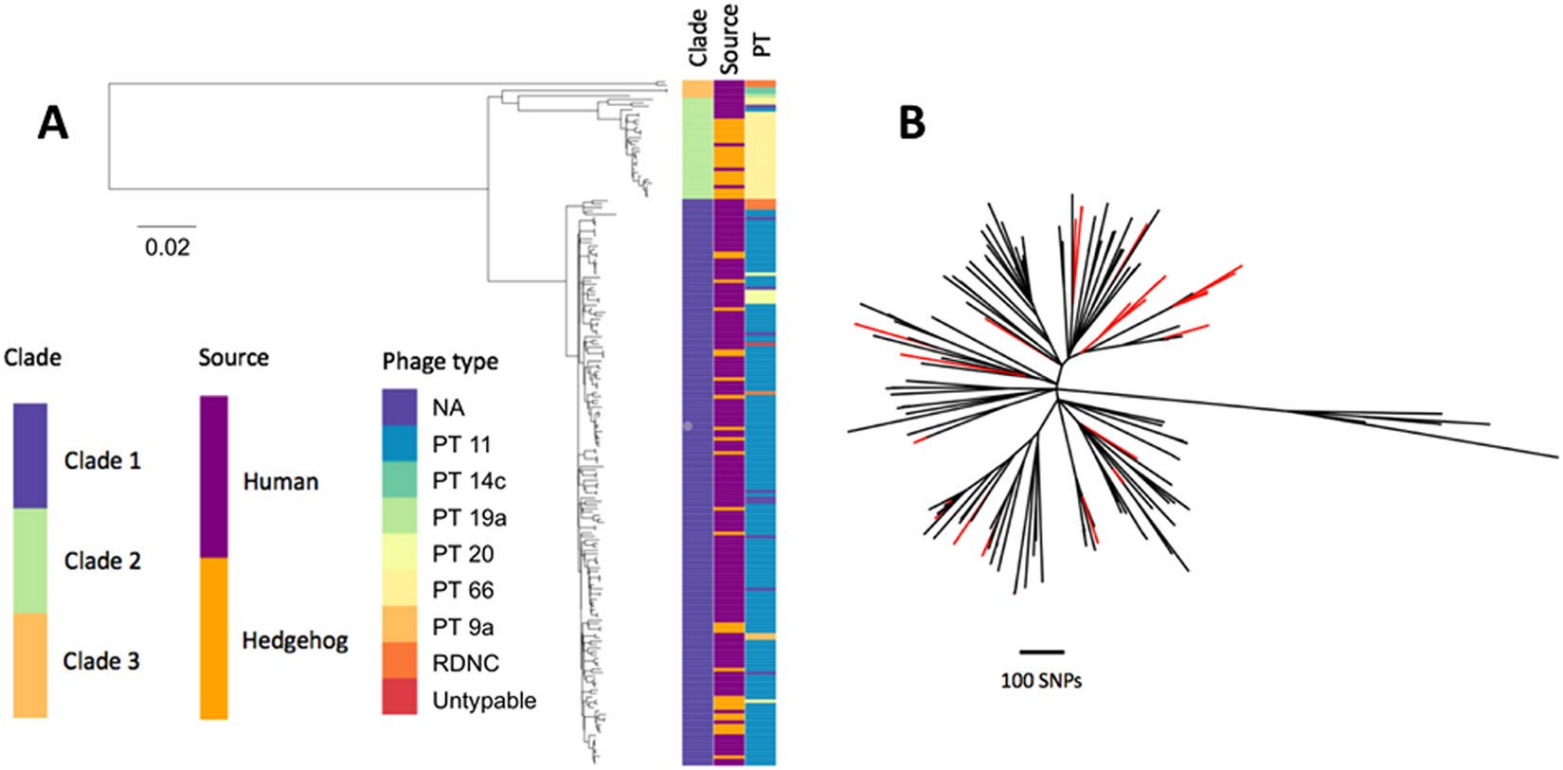
Whole genome sequence single nucleotide polymorphism phylogenetic analysis of ST183 isolates of *Salmonella* Enteritidis. (**A**) All ST183 isolates. (**B**) Focus on the PT11 clade, branches where the majority of descendants are hedgehogs are coloured red.

There was minimal impact of recombination on the phylogeny, with a recombination/mutation (r/m) of 0.06 as determined by Gubbins. A whole genome SNP based phylogeny based on the 5880 variable positions showed two main clades within ST183 (Fig. 4). Clade 1 contains 162 isolates, of which the majority of those for which phage type was available were PT11 isolates (141/153). Clade 2 contains 29 isolates, of which the majority of those for which phage type was available were PT66 isolates (27/28). Both clades contained human and hedgehog isolates and within the lineages, isolates did not phylogenetically cluster by host species (Fig. 4).

**Figure 5:**
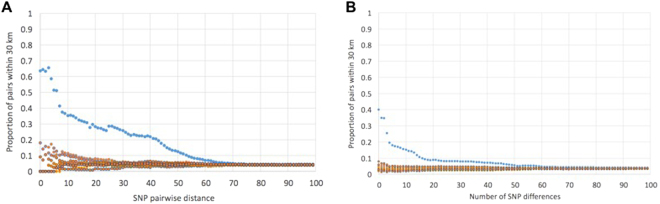
Relationship between genetic and geographic distance for (**A**) ST183 and (**B**) ST11 *Salmonella* Enteritidis. For an increasing threshold of SNP distances, the proportion of pairs with a SNP distance less than or equal to that threshold, that were within 30 km straight line distance was calculated. To aid interpretation, we give the following example, of all the pairs of ST183 that were within 40 SNPs or less of each other, 22% were geographically located within 30 km of each other. For both control and ST183, the data was shuffled randomly 10 times, these data are plotted in orange, non-shuffled data are in blue.

The distribution of pairwise SNP data was consistent with the epidemiological data that indicated there have been no outbreaks of ST183. Isolates of *Salmonella* originating from the same epidemiological event were almost always closely related (<25 SNPs apart)^33–37^. We hypothesised that there are differences between ST11 and ST183 in terms of the events resulting in infection, therefore it is most relevant to look at the pairwise SNP distance in this approximate range (~25 SNPs). The distribution of pairwise SNP differences (just for pairs within ≤40 SNPs, as this is the relevant distance in terms of outbreaks) was compared between ST183 and ST11 (which accounts for the majority of known *S*. Enteritidis isolates and can be considered ‘typical’ *S*. Enteriditis in humans) and there were marked differences (Supplementary Figure S1). There was a bimodal distribution in ST11, with peaks at 0–5 SNPs and 14–17 SNPs. However, the ST183 data had no peak at 0–5 SNPs, which is consistent with the lack of outbreaks of this ST with all human ST183 infections considered sporadic. Of the pairs of ST183 with ≤40 SNPs pair wise distance, 78% (95% CI 77–79%) of them fell in the 25–40 SNP distance range, compared with 23% (95% CI 23–23%) of ST11 in the same range. The ‘control’ set which was subsampled from ST11 for the phylogeographical analysis (i.e. Figure 5), was representative of the entirety of ST11.

Compared with PT66, which showed strong geographic restriction, there was a subtle, but detectable, geographical signal across all human ST183 isolates (Fig. 5A). Only human isolates were used for this analysis because PHE samples uniformly across England and Wales based on reported *Salmonella* cases. In contrast, the hedgehog isolates were a convenience sample, combining syndromic surveillance at a specific wildlife centre and scanning surveillance, therefore potential for spatial bias in sampling exists. Of all pairs of human derived ST183 that were within 40 SNPs of each other, 22% were from within 30 km of each other. Conversely, for a ‘control’ set of ST11 (Fig. 5B), of the pairs of isolates that were within 40 SNPs of each other 7% of isolates were from within 30 km. The spatio-genetic relationship within the ‘control’ group of ST11 was driven by cases from a known outbreak that were epidemiologically linked to food consumption at the same restaurant^36,38^. The stronger spatio-genetic relationship within the ST183 was not driven by distinct epidemiological events such as point source outbreaks. The tendency for phylogenetically related ST183 isolates to be from close geographic proximity can also be seen in phylogenetic trees annotated with the PHE centre each isolate came from (Supplementary Figure S2a), compared with the ‘control’ set of *S*. Enteritidis (Supplementary Figure S2b).

The incidence of ST11 was approximately the same in rural and urban settings (3.52 and 3.71 cases per 100 000 people; number of people in each setting obtained from the 2011 ONS census information), while the incidence of ST183 was almost double in rural settings (0.35 cases per 100 000 people in rural settings compared with 0.18 cases per 100 000 people in urban settings) (Table 1). These numbers were used to calculate an odds ratio which showed that people living in a rural setting had a significantly higher chance of being infected with ST183 than those in urban settings (OR 2.05, 95% CI 1.4–3.1, p = 0.0006).

**Table 1:** Frequency of *Salmonella* Enteritidis ST183 and ST11 in rural and urban households in 2015.

	ST183 - frequency	ST183 - incidence (per 100 000 people)	ST11 - frequency	ST11 - incidence (per 100 000 people)
Rural	36	0.35	364	3.52
Urban	82	0.18	1696	3.71

### Demographic summary of human *Salmonella* infections

**Figure 6:**
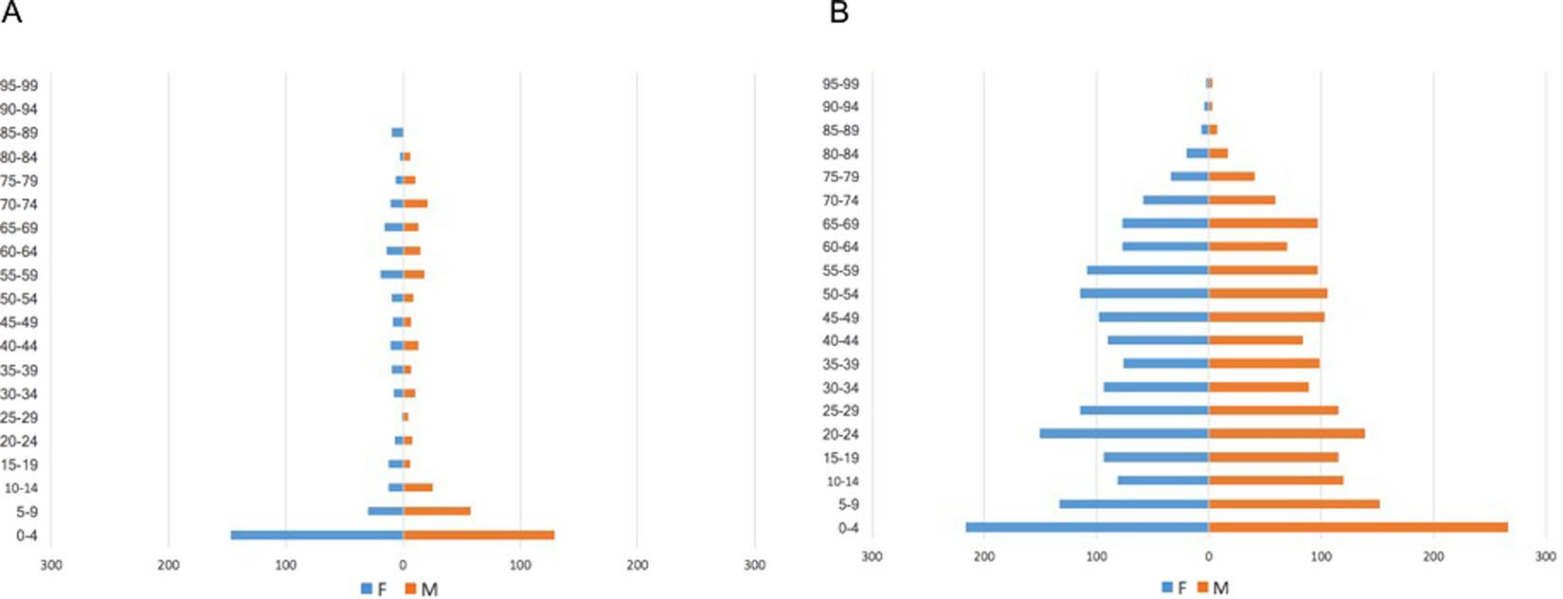
Age-sex distribution of human (**A**) PT11 and (**B**) non-PT11 infections, in 2015.

Descriptive epidemiology was carried out for all 696 human *S*. Enteritidis PT11 in the PHE database for which a full year of data was available (2004–2015). In terms of the demography of the human population suffering from PT11 infections (Fig. 6A) compared with 3435 non-PT11 Enteritidis infections received by PHE between July 2014 and March 2016 (Fig. 6B), a marked difference in affected populations was apparent; 40% of PT11 infections occurring in the 0–4 infant age group, compared with 14% for non-PT11 Enteritidis. Children aged 4 or under were significantly more likely to be infected with ST183 compared with their likelihood of infection with ST11 (OR = 4, 95% CI 3.4–4.8, p < 0.0001). The majority of human *S*. Enteritidis PT11 and all PT66 isolates were derived from faecal samples and the remaining 1.7% from blood samples.

### Retrospective data review

Available PHE databases (2004–2015 inclusive) include 800 *S*. Enteritidis PT11 isolates, 719 of which were from people and 32 from hedgehogs (including 4 isolates from hedgehogs 2010–2012 which were not part of this study). Fourteen *S*. Enteritidis PT11 isolates were from other non-human hosts and comprised livestock (chicken 1, cattle 2), companion animals (cat 2, dog 2, horse 4, guinea pig 1) and two wild birds. *S*. Enteritidis ST183 PT66 isolates have been isolated from 7 people (4 from Scotland, 3 from England) and 20 hedgehogs from Scotland in this study and no other hosts.

Available APHA databases (2002–2016 inclusive) include a total of 77 *S*. Enteritidis PT11 isolates. All wildlife isolates were from hedgehogs (25), collected from 11 of the 15 years of the monitored period, with the exception of a single isolate from a wild bird (swallow of unidentified sp.). The other species comprised livestock (horse 14, cattle 4, chicken 3, sheep 2), companion animals (dog 15, cat 7, 1 guinea pig) and zoo/mixed species (5).

### Antimicrobial resistance data

Antimicrobial resistance (AMR) data were available for 47 isolates of *S*. Enteritidis from hedgehogs (46 from this study and one from the APHA archive). Of these, 26 were PT11 and 21 were PT66, all were sensitive to the antimicrobials tested and no genotypic resistance determinants were identified.

For the period January 2006–December 2015, AMR data was available for 74% (441) of the human *S*. Enteritidis PT11 isolates. Of these, eight isolates showed some AMR: two were resistant to ampicillin, one to ampicillin and chloramphenicol, one to gentamicin, one to sulphonamide, one to trimethoprim, one to tetracycline and one to sulphonamide, spectinomycin and tetracycline. Antimicrobial resistance data for human isolates of *S*. Enteritidis PT66 was available for one isolate from England and Wales and three isolates from Scotland. All four isolates were found to be fully sensitive to the antimicrobials tested.

In terms of human isolates for which WGS data was available, there was phenotypic antimicrobial resistance data for 37 isolates, of which 23 were PT11, 4 were PT66, 1 was PT20 and PT was undetermined for 9. All these isolates were sensitive phenotypically, and no genotypic resistance determinants were identified. Of the 111 human isolates with WGS for which phenotypic information was not available, there were no resistance determinants detected for 109 of them. Two isolates, both outliers (i.e. not in the PT66 or PT11 clades) carried genes encoding the same resistance determinants – blaTEM-1, *strA*, *strB* and *sul2*. blaTEM-1 confers resistance to beta-lactam antibiotics, *strA* and *strB* confer resistance to streptomycin and *sul2* confers resistance to sulphonamides.

## Discussion

Phylogenetic analysis of the hedgehog *S*. Enteritidis isolates and of matched biotypes available from humans in GB demonstrated that PT11 and PT66 formed two distinct clades within ST183, with high, but not total, congruence between the phage types and phylogenetic groupings.

PT11 infection in hedgehogs was confirmed with a widespread distribution in England and Scotland whilst PT66 was only isolated from southern and central Scotland: the extent to which the spatial distribution of PT66 confirmation is biased with the selective strategy of NWRC post-mortem examination submissions requires further investigation. Biotyping of *S*. Enteritidis isolates derived from examination of hedgehog faecal samples supported the same spatial distributions as the carcass investigations. WGS examination of the variant PT15 isolates from archived isolates at the reference laboratories in APHA and Stobhill were also consistent with this pattern.

The pathological findings of hedgehogs with PT11 infection varied: some had evidence of gastrointestinal disease whilst others had evidence of unrelated disease. Evidence of salmonellosis was present in 26% of PT11 hedgehogs, but the significance of *Salmonella* infection was uncertain in the remaining animals. Both juvenile and adult age classes were affected. These findings are consistent with those of Keymer *et al*.^12^, who found evidence of disease (i.e. enteritis, septicaemia) and also clinically inapparent infection with PT11. The wide spatial distribution of PT11 hedgehogs supports the hypothesis of Keymer *et al*.^12^ that it is endemic in hedgehogs in GB.

PT66 isolation was consistently associated with severe lesions and salmonellosis in adult hedgehogs. Whether inapparent infection or other disease occurs with PT66 is unclear as our study specifically targeted animals with MLN enlargement from the region where this PT was found. The presentation in some hedgehogs was striking with the MLN enlargement accounting for up to 11.5% of body weight. Data on hedgehog disease is limited but, to the authors’ knowledge, this presentation has not previously been reported. This might be due to recent emergence of this novel biotype. As mentioned previously, further scanning surveillance is required to investigate the spatial distribution of PT66 infection and its range of disease presentations in order to overcome potential bias through the sampling strategy employed in this study. Combining the results of scanning surveillance with population monitoring will help to indicate if there is any impact of this novel phage type on the hedgehog population.

The narrow time period (2012–2014) from which PT66 isolates are available for WGS precludes accurate application of molecular clock techniques to estimate the date of emergence of this biotype. The NWRC first noted the syndrome of hedgehog casualty presentation with markedly enlarged MLNs in 2008: disease investigation in 2012 provided preliminary microbiological evidence of a link with *Salmonella* infection, which prompted the current study. The NWRC noted an increase in hedgehog submissions with this presentation in 2011 and 2012 (authors’ unpublished observations), but this was coincident with an overall increase in hedgehog admissions, therefore it is not possible to assess whether this represents a recent emergence event or simply raised awareness.

Whilst the number of *Salmonella* culture-positive faecal samples with a description of the faecal appearance was low, the majority (4/5) were abnormal in appearance, which may be consistent with enteritis and diarrhoea due to salmonellosis. However, numerous alternative explanations are possible and without further clinical or pathological investigations of these hedgehogs the significance of infection cannot be assessed. The prevalence of enteric *Salmonella* infection of 3% (7/208) compares with 10% (9/90) of hedgehog faeces screened in a wildlife rehabilitation centre in Germany^11^. Whilst the samples were collected within 48 hours of admission to centres in GB, information on the duration in captivity was not available in the German study, therefore increased prevalence due to nosocomial infection cannot be excluded for that cohort.

Review of available SRS and APHA databases indicate that the hedgehog is the non-human species from which PT11 is most frequently isolated in GB and accounts for all recoveries from wild animals with the exception of three incidents from wild birds. PT11 was the most frequent biotype isolated from hedgehogs in the current study. This is consistent with the hedgehog being the principle reservoir of PT11 infection^12^. PT11 was isolated from small numbers of companion animal and livestock species in GB; however, these represent a minority of *Salmonella* phage types from these hosts^39^. Sporadic infection of livestock and companion animals with PT11 has also been recorded in continental Europe^13^. A similar pattern has been observed in passerine salmonellosis where the vast majority of infections are accounted for by a small number of apparently host-adapted biotypes (e.g. *S*. Typhimurium definitive type (DT)40 and DT56v). However, the wild bird-associated biotypes are not host restricted and infection has been shown to account for a minority of infections in livestock, companion animals and people^6,40^.

The WGS SNP phylogenetic analyses found no strong association between phylogeny and host species for ST183, suggesting inter-species transmissions. Even within an ideal sampling frame, it is difficult to infer the direction of transmission between humans and animals through WGS data^41^. The sample is biased in this study with data available from many more human than hedgehog isolates, which might indicate anthroponotic infection of hedgehogs. Human surveillance, however, is much greater than hedgehog surveillance, yet PT11 infection of hedgehogs is found frequently and with a wide distribution and PT11 is the predominant *Salmonella* in hedgehogs, but not people. These findings are more consistent with a wildlife source of infection.

Both human and hedgehog PT11 infections were widespread across GB. Epidemiologically related pairs (i.e. same point source exposure such as with food-borne infection) of *Salmonella* spp. are typically within 5 SNPs of each other^33–35^. In our study, this was seen in the distribution of pairwise SNP distances for ST11. However, for ST183, there were a very small number of pairs with a distance of <5 SNPs, indicating a lack of human cases sharing a point exposure. Marked spatial structuring was observed in human ST183 isolates, with those more geographically proximate being most phylogenetically related. As human infections due to ST183 were considered sporadic, as opposed to outbreaks, this structuring indicates a local source of infection, for example a wildlife reservoir, rather than alternative sources such as foreign travel or food.

Human ST183 infections were significantly more likely to occur in rural than urban settings, which also supports infection from a wildlife reservoir. Whilst hedgehogs do utilise domestic gardens across habitat types, they are typically abundant in farmland and hedgerows in rural habitats^42^. Whilst screening of terrestrial wild mammal and birds has found no evidence of an alternate wild species as a reservoir host for ST183, this possibility cannot be excluded.

A significantly higher proportion of human ST183 infections were in infants compared with ST11, an observation which was previously made in GB and continental Europe^12–14^. Notably, a similar marked age skew was seen in the demography of human infections with passerine-associated phage types of S. Typhimurium also believed to be contracted from a wildlife reservoir: it was postulated that this could relate in part due to poor hygiene of children and increased risk of exposure through outdoor play^6^. These findings support an environmental source of infection with PT11, as opposed to a food source which has a demographic distribution similar to that for ‘normal’ *S*. Enteritidis ST11.

No evidence of AMR was present in PT11 from hedgehogs, the vast majority of PT11 from humans and PT66 from both species. Considering that SRS data indicate some evidence of AMR in circa 30% of *S*. Enteritidis isolates of all biotypes from across hosts (*unpublished data*), this is quite unusual. It likely reflects the environmental nature of PT11.

Epidemiological studies in continental Europe have identified contact with hedgehogs as a common exposure risk for human *Salmonella* infection^14^. Our study supports the hedgehog being a potential source of infection. Direct and indirect human-hedgehog contact is common in GB, with supplementary feeding regularly practised by some householders^43^. Whilst our study supports zoonotic infection with ST183 of humans from hedgehogs, the public health risk must be viewed in context. In total, PT11 accounted for only 0.6% of reported human *Salmonella* infections, over the period January 2006–December 2015 in England and Wales and 1.6% of the *S*. Enteritidis isolates during this 10 year period.

To reduce risks to people and wildlife, sensible hygiene practices are required when provisioning wild animals^6^. Avoiding direct contact with hedgehogs or their faeces, and washing hands after feeding hedgehogs or outdoor play, are recommended to reduce the risk of exposure. Any public health risk can be further mitigated by raising the public, veterinary and medical communities’ awareness of *Salmonella* infection in hedgehogs.

## Electronic supplementary material

Supplementary Information

Supplementary Worksheet 1
