# Supplementary material for: Salmonella Enteritidis ST183: emerging and endemic biotypes affecting western European hedgehogs (Erinaceus europaeus) and people in Great Britain
Source: Sci Rep. 2018 Feb 5;8:2449. doi: 10.1038/s41598-017-18667-2 (PMC5799193; doi:10.1038/s41598-017-18667-2)
Supplement: Supplementary file 1 — Supplementary Information [file 41598_2017_18667_MOESM1_ESM.pdf]

Salmonella Enteritidis ST183: emerging and endemic biotypes affecting western European hedgehogs (_Erinaceus europaeus_) and people in Great Britain

Becki Lawson[1], Lydia H.V. Franklinos[1,5], Julia Rodriguez-Ramos Fernandez[1,6], Clare Wend-Hansen[2], Satheesh Nair[2], Shaheed K. Macgregor[1], Shinto K. John[1], Romain Pizzi[3], Alejandro Núñez[4], Philip M. Ashton[2], Andrew A. Cunningham[1] & Elizabeth M. de Pinna[2]

SUPPLEMENTARY MATERIALS

Supplementary Methods S1

Microbiology
Tissue samples were plated directly onto (1) Colombia blood agar supplemented with 5% horse blood (QCM Laboratories, London, UK) in triplicate, and incubated under each of aerobic, anaerobic, and microaerophilic conditions; (2) xylose-lysine deoxycholate (XLD) agar (QCM Laboratories) and incubated under aerobic conditions; (3) chocolate blood agar (QCM Laboratories) and incubated under 5–10% CO2 conditions; and (4) immersed in selenite Salmonella-selective enrichment broth (QCM Laboratories) under aerobic conditions for 24 hours followed by subculture onto XLD agar aerobically. All samples were incubated at 37 ^{o}C with inspection of the agar plates at 24, 48, and 120 hours post-inoculation.

Supplementary Methods S2

Immunohistochemistry
Immunohistochemistry (IHC) was conducted on tissues from a subset of hedgehogs examined to confirm co-location of _Salmonella_ sp. with microscopic lesions. _Salmonella_ spp. antigen was detected by IHC in 4 micron thick formalin-fixed paraffin wax embedded tissue sections using an automated protocol optimised for use on the Ventana Discovery XT staining module (Ventana Medical Systems, Tucson, Arizona, USA). Tissue sections were dewaxed, prior to the application of BacTrace® goat anti-Salmonella CSA-1 antibody (KPL, Gaithersburg, Maryland, USA) diluted 1:200 in Ventana Ab Diluent (Ventana Medical Systems, USA) for 60 minutes at room temperature. Antibody-antigen interaction was detected using a rabbit anti-goat Ig antibody (6 µg/ml, Vector Laboratories Peterborough, United Kingdom) diluted in Ventana Ab diluent (Ventana Medical Systems, USA) followed by OMap anti-Rabbit HRP (multimer HRP, Ventana Medical Systems, USA) and then visualised using the ChromoMap DAB kit (Ventana Medical Systems, USA). Sections were then counterstained in haematoxylin (Ventana Reagents, Tucson, Arizona, USA) for 12 minutes, before being permanently mounted for interpretation. Concentration-matched antibody goat IgG isotype (R&D Systems, Minneapolis, Minnesota, USA) in addition to known positive tissues were included as a test control. The specificity of the immunohistochemical technique had been validated in tissues containing a variety of enterobacteria without detection of relevant cross reaction.

Supplementary Methods S3

Antimicrobial panel tested in antimicrobial resistance studies based on Nair et al (2016)[20].
Susceptibility testing was performed by a breakpoint method using Iso-sensitest agar or Muller-Hinton agar. The antimicrobial concentrations used were: ampicillin 8 mg/L, chloramphenicol 8 and 16 mg/L, colistin 2 mg/L, sulphonamide 64 mg/L, gentamicin 2 mg/L, tobramycin 2 mg/L, amikacin 8 mg/L, streptomycin 16 mg/L, tetracycline 8 mg/L , trimethoprim 2 mg/L , nalidixic acid 16 mg/L, ciprofloxacin 0.064 and 0.5 mg/L, ceftazidime 1 and 2 mg/L, cefotaxime 0.5 and 1 mg/L, cefoxitin 8 mg/L, cefpirome 1 mg/L, ertapenem 0.064 and 0.5 mg/L, and temocillin 128 mg/L.

Breakpoints were based on those recommended by EUCAST, EFSA http://ecdc.europa.eu/en/publications/Publications/AMR-salmonella-campylobacter-protocol-monitoring.pdf and EU Reference Laboratory Antimicrobial Resistance recommended screening guidance http://www.crl-ar.eu/201-resources.htm#cutoff.

Supplementary Methods S4

DNA extraction and whole genome sequencing

DNA extraction of Salmonella isolates was carried out using a modified protocol of the Qiasymphony DSP DNA midi kit (Qiagen). In brief, 0.7 ml of overnight Salmonella culture in a 96 deep well plate was harvested. Bacterial cells were pre-lysed in 220 µl of ATL buffer (Qiagen, UK) and 20 µl Proteinase K (Qiagen), and incubated shaking for 30 min at 56 °C. Four µl of RNase at 100 mg/ml (Qiagen) was added to the lysed cells and re-incubated for a further 15 minutes at 37 °C. This step increases the purity of the DNA for further downstream sequencing. Extraction of DNA from the treated cells was performed on the Qiasymphony SP platform (Qiagen) and eluted in 100 µl of water. DNA concentration using the GloMax system (Promega, UK) was determined for the following sequencing steps.

Extracted DNA was then processed using the NexteraXT sample preparation method and sequenced with a standard 2x101 base protocol on a HiSeq 2500 Instrument in fast mode (Illumina, San Diego, CA, USA).

Supplementary Figure S1

The distribution of pairwise SNP distances of 196 ST183 (hedgehog associated), 2060 ST11 ('typical' *S*. Enteritidis) and 71 'control' ST11.

ST11 as a whole had a bimodal distribution with peaks at 0-5 SNPs and 14-17 SNPs, with the peak at 0-5 SNPs representing probable shared point source exposures. Due to the computational time required for permutation analysis (i.e. Figure 5), a subset of ST11 control data set was taken. ST183 had no peak at 0-5 SNPs, representing the lack of outbreaks associated with this ST. A larger proportion of the ST183 isolates had pairwise SNP distances of 25-40.

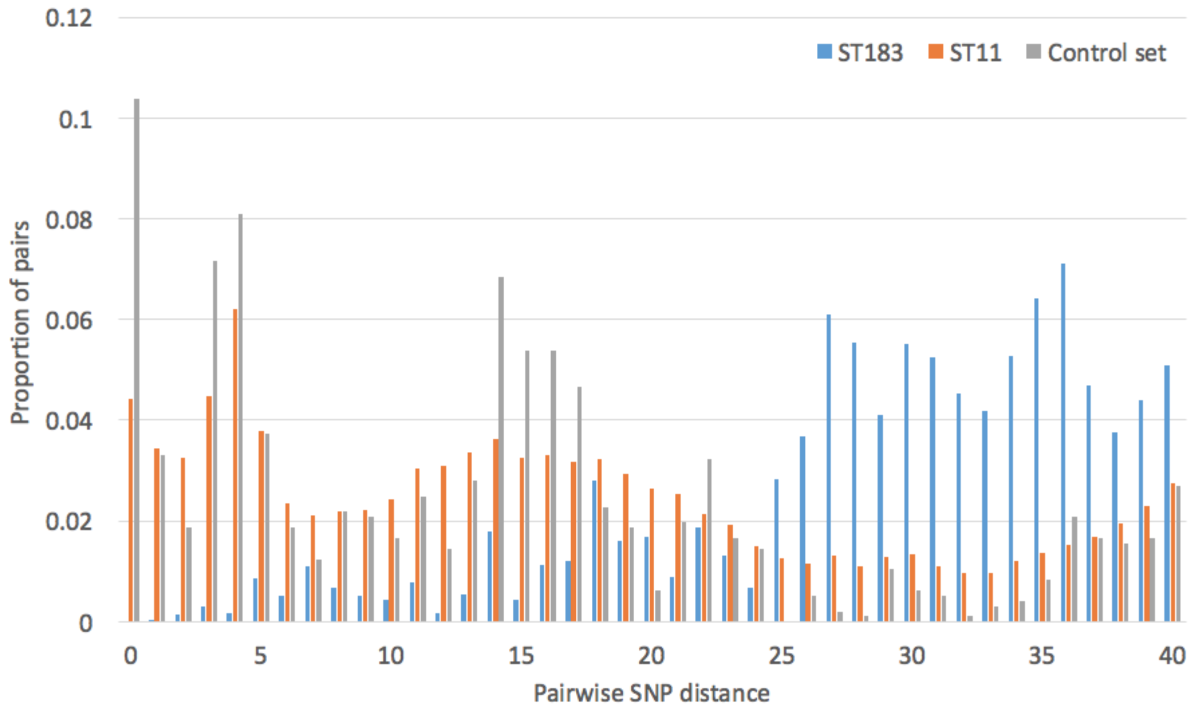

Supplementary Figure S2

a. ST183 *Salmonella* Enteritidis maximum likelihood phylogeny annotated with geographical region built with 5881 variant positions. Regions with more than 5 isolates have been given the same colour for clarity.

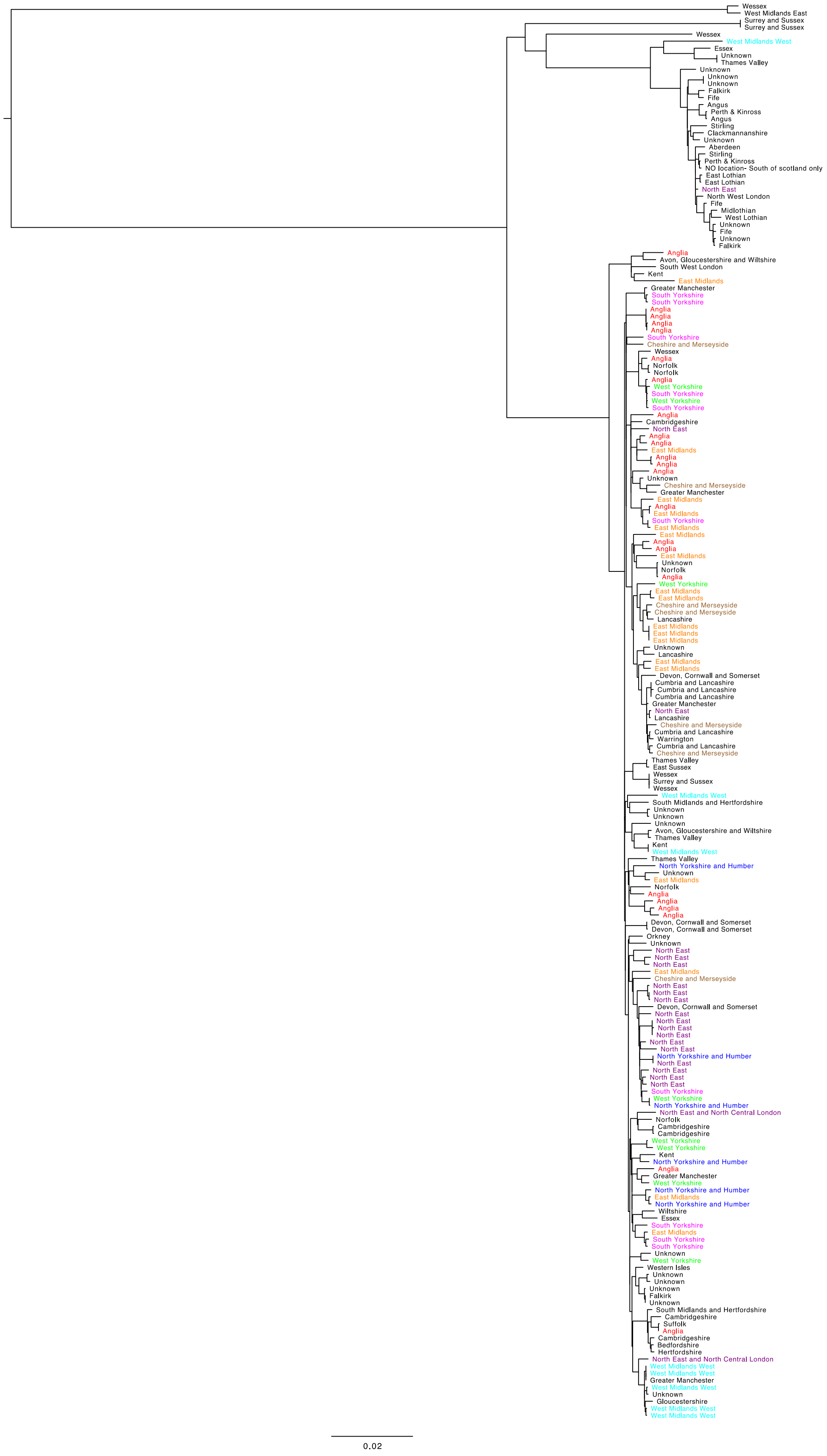
Wessex
West Midlands East
Surrey and Sussex
Surrey and Sussex
Wessex
West Midlands West
Essex
Unknown
Thames Valley
Unknown
Unknown
Unknown
Falkirk
Fife
Angus
Perth & Kinross
Angus
Stirling
Clackmannanshire
Unknown
Aberdeen
Stirling
Perth & Kinross
NO location- South of scotland only
East Lothian
East Lothian
North East
North West London
Fife
Midlothian
West Lothian
Unknown
Fife
Unknown
Falkirk
Anglia
Avon, Gloucestershire and Wiltshire
South West London
Kent
East Midlands
Greater Manchester
South Yorkshire
South Yorkshire
Anglia
Anglia
Anglia
Anglia
South Yorkshire
Cheshire and Merseyside
Wessex
Anglia
Norfolk
Norfolk
Anglia
West Yorkshire
South Yorkshire
West Yorkshire
South Yorkshire
Anglia
Cambridgeshire
North East
Anglia
Anglia
East Midlands
Anglia
Anglia
Anglia
Unknown
Cheshire and Merseyside
Greater Manchester
East Midlands
Anglia
East Midlands
South Yorkshire
East Midlands
East Midlands
Anglia
Anglia
East Midlands
Unknown
Norfolk
Anglia
West Yorkshire
East Midlands
East Midlands
Cheshire and Merseyside
Cheshire and Merseyside
Lancashire
East Midlands
East Midlands
East Midlands
Unknown
Lancashire
East Midlands
East Midlands
Devon, Cornwall and Somerset
Cumbria and Lancashire
Cumbria and Lancashire
Cumbria and Lancashire
Greater Manchester
North East
Lancashire
Cheshire and Merseyside
Cumbria and Lancashire
Warrington
Cumbria and Lancashire
Cheshire and Merseyside
Thames Valley
East Sussex
Wessex
Surrey and Sussex
Wessex
West Midlands West
South Midlands and Hertfordshire
Unknown
Unknown
Unknown
Avon, Gloucestershire and Wiltshire
Thames Valley
Kent
West Midlands West
Thames Valley
North Yorkshire and Humber
Unknown
East Midlands
Norfolk
Anglia
Anglia
Anglia
Anglia
Devon, Cornwall and Somerset
Devon, Cornwall and Somerset
Orkney
Unknown
North East
North East
North East
East Midlands
Cheshire and Merseyside
North East
North East
North East
Devon, Cornwall and Somerset
North East
North East
North East
North East
North East
North East
North Yorkshire and Humber
North East
North East
North East
North East
South Yorkshire
West Yorkshire
North Yorkshire and Humber
North East and North Central London
Norfolk
Cambridgeshire
Cambridgeshire
West Yorkshire
West Yorkshire
Kent
North Yorkshire and Humber
Anglia
Greater Manchester
West Yorkshire
North Yorkshire and Humber
East Midlands
North Yorkshire and Humber
Wiltshire
Essex
South Yorkshire
East Midlands
South Yorkshire
South Yorkshire
Unknown
West Yorkshire
Western Isles
Unknown
Unknown
Unknown
Falkirk
Unknown
South Midlands and Hertfordshire
Cambridgeshire
Suffolk
Anglia
Cambridgeshire
Bedfordshire
Hertfordshire
North East and North Central London
West Midlands West
West Midlands West
Greater Manchester
West Midlands West
Unknown
Gloucestershire
West Midlands West
West Midlands West
0.02

b. Control *Salmonella* Enteritidis maximum likelihood phylogeny annotated with geographical region built with 950 variants positions. Regions with more than 3 isolates have been given the same colour for clarity.

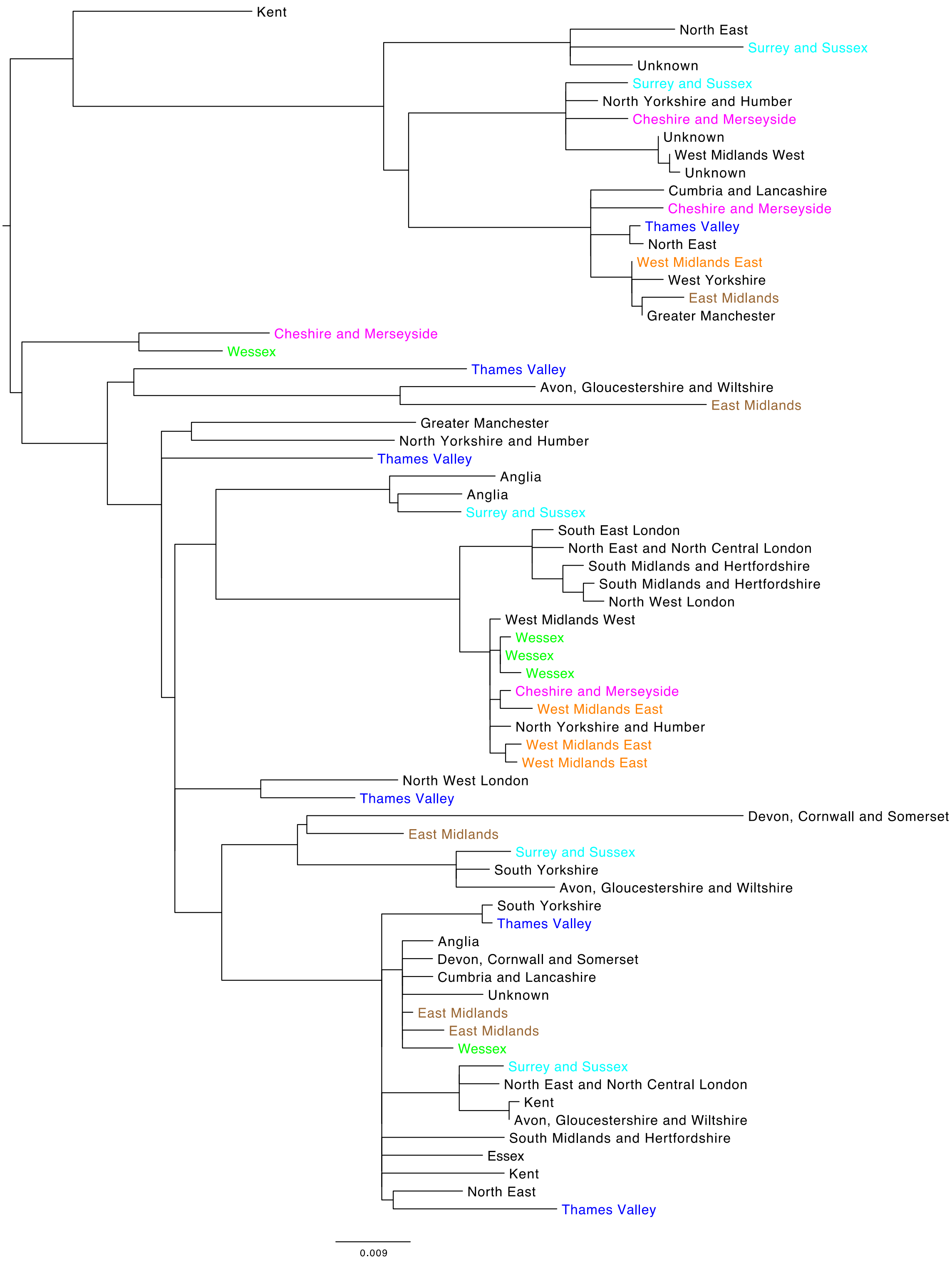

Supplementary Table S1: Summary of signalment and macroscopic abnormalities in hedgehogs with *Salmonella* Enteritidis PT11 infection

Case number	Date and location	Signalment	Body condition	Body weight (g)	Tissues sampled for microbiology*	Significant macroscopic abnormalities	MLN weight (g)	MLN dimensions (length x width x breath (mm))	Confirmed/ suspected cause of death
XT0717-12	Aug 2012; Lancashire, England	Male Juvenile	Thin	108	Liver*, SI contents, lung, brain	Pulmonary and intra-cranial haemorrhage.	-	-	Trauma<br><br>(*S*. Enteritidis PT11 unknown significance)
XT0719-13	Nov 2011; Hertfordshire, England	Male Juvenile	Thin	169	Liver, SI contents*, MLN	Multifocal haemorrhage in the soft tissues of sternal region. Haemothorax. Enlarged MLN x ≥2.	-	10 x 8 x 5	Trauma<br><br>(*S*. Enteritidis PT11 unknown significance)
XT0754-13	Nov 2012; Cambridgeshire, England	Female Juvenile	Thin	259	Liver*, SI contents*, lung*	Parasitic pneumonia.	-	-	Infectious disease; parasitic pneumonia<br><br>(*S*. Enteritidis PT11 unknown significance)
XT0767-13	Nov 2012; Cambridgeshire, England	Female Juvenile	Thin	213	Liver*, SI contents*, lung*	Oesophagitis and gastritis. Enlarged MLN x ≥2. Parasitic pneumonia.	-	8 x - x -	Infectious disease; parasitic pneumonia<br><br>(*S*. Enteritidis

									PT11 unknown significance)
XT0782-13	Dec 2012; Hertfordshire, England	Female Juvenile	Thin	208	Liver*, SI contents*, urine*, lung	Pneumonia.	-	-	Infectious disease; pneumonia<br><br>(*S*. Enteritidis PT11 unknown significance)
XT0036-14	Aug 2013; Falkirk, Scotland	Female Adult	Normal	549	Liver*, SI contents*, MLN*, LI contents*, skin, heart, pleural cavity	Abscessated MLN. Fibrinous inflammation of the abdominal cavity with peritoneal adhesions. Enteritis.	30.8	41 x 53 x 29	Euthanasia + Infectious disease; salmonellosis
XT0443-14[H]	Apr 2014; Norfolk, England	Male Subadult	Normal	404	Liver*, SI contents, MLN*	Conjunctivitis. Parasitic pneumonia. Enlarged MLN.	-	-	*Refer to histopathological findings*
XT0480-14	Apr 2014; Orkney, Scotland	Male Adult	Thin	329	Liver*, SI contents*	-	-	-	Undetermined<br><br>(*S*. Enteritidis PT11 unknown significance)
XT0881-14[C,H]	Apr 2012; Fife, Scotland	Male Adult	Normal	585	Liver, SI contents, MLN*, skin, LI LN abscess*, urine	Abscessated MLN x ≥ 2. Acanthocephalans in peritoneal cavity.	114.1	70 x 48 x -	*Refer to histopathological findings*
XT0995-14[H]	Aug 2014; Norfolk, England	Female Juvenile	Emaciated	160	Liver, SI contents*, MLN*, kidney*, skin	Pyelonephritis. Parasitic pneumonia. Gastritis (gastric ulceration).	-	-	*Refer to histopathological findings*
XT1207-14[H]	Nov 2013; Cambridgeshire, England	Male Adult	Emaciated	289	Liver*, SI contents*, MLN*, lung*, skin	Abscessated MLN. Parasitic pneumonia. Severe periodontal disease.	-	51 x 26 x 9	*Refer to histopathological findings*

XT0107-15	Sep 2014; Cambridgeshire, England	Male Adult	Thin	186	Liver*, SI contents*, MLN*, throat, skin	Haematoma in subcutis.	-	-	Undetermined<br><br>(*S*. Enteritidis PT11 unknown significance)
XT0270-15	April 2015; East Sussex, England	Female Juvenile	Emaciated	309	Liver*, SI contents*, MLN*, throat, skin	High Ixodid sp. tick burden.	-	-	Undetermined<br><br>(*S*. Enteritidis PT11 unknown significance)
XT0541-15	Sep 2014; Cambridgeshire, England	Female Juvenile	Thin	366	Liver*, SI contents*, MLN*, lung*, skin	Enteritis. Parasitic pneumonia. Mild otitis externa.	-	-	Infectious disease; salmonellosis + parasitic pneumonia
XT0727-15	Jul 2014; Cambridgeshire, England	Female Juvenile	Normal	136	Liver*, SI contents*, MLN*, throat, skin	Dermatitis. Parasitic pneumonia.	-	-	Infectious disease; parasitic pneumonia<br><br>(*S*. Enteritidis PT11 unknown significance)
XT0859-15	Jul 2015; Lancashire, England	Female Adult	Normal	426	Liver*, SI contents*, MLN*, throat, skin	Abscessated MLN. High Ixodid sp. tick burden. Possible rhinitis. Possible gastritis.	-	-	Infectious disease; salmonellosis
XT0877-15	Jul 2015; Wiltshire, England	Male Juvenile	Normal	287	Liver*, SI contents*, MLN*, throat, skin	Gastritis (possible gastric ulceration). Enlarged MLN x ≥2.	-	-	Infectious disease; salmonellosis
XT1054-15	Aug 2015; Buckinghamshire, England	Male Juvenile	Emaciated	211	Liver*, SI contents*, MLN*, throat, skin	Skull fractures. Parasitic pneumonia.	-	-	Trauma<br><br>(*S*. Enteritidis PT11 unknown

									significance)
XT1094-15[H]	Aug 2015; Western Isles, Scotland	Female Adult	Normal	604	Liver*, SI contents*, MLN*, throat, skin	Axillary abscess. Abscessated MLN x ≥2. Parasitic pneumonia.	10.4	39 x 20 x 14	*Refer to histopathological findings*
XT1196-15	Sep 2015; Hertfordshire, England	Male Subadult	Emaciated	325	Liver*, SI contents*, MLN*, throat, skin	Haemorrhage in subcutis. Ascites.	-	35 x 7 x 7	Euthanasia + undetermined/ emaciation<br><br>(*S*. Enteritidis PT11 unknown significance)
XT1270-15[H]	Oct 2014; Bedfordshire, England	Female Adult	Thin	490	Liver*, SI contents*, MLN*, throat, skin	Periodontal disease. Parasitic pneumonia.	-	-	*Refer to histopathological findings*
XT1293-15	Sep 2015; Lancashire, England	Male Juvenile	Emaciated	360	Liver*, SI contents*, MLN*, throat, skin	Parasitic pneumonia. High Ixodid sp. tick burden.	-	60 x 10 x 10	Infectious disease; parasitic pneumonia<br><br>(*S*. Enteritidis PT11 unknown significance)
XT1315-15[H]	Oct 2015; Lancashire, England	Male Adult	Normal	306	Liver*, SI contents*, MLN*, throat, skin	Fractures to ribs, cervical vertebrae, skull and tibia. Severe internal haemorrhage. Hepatic rupture. Parasitic pneumonia. High Ixodid sp. tick burden.	1.2	-	Severe trauma<br><br>(*S*. Enteritidis PT11 unknown significance)
XT1341-15	Oct 2015; Norfolk, England	Male Juvenile	Normal	204	Liver*, SI contents*, MLN*, throat, skin	Parasitic pneumonia and oedema.	-	50 x 15 x 3	Infectious disease; parasitic pneumonia

									(*S*. Enteritidis PT11 unknown significance)
XT1351-15	Nov 2015; Lancashire, England	Male Juvenile	Thin	100	Liver*, SI contents*, MLN*, throat, skin	Parasitic pneumonia. Possible gastritis and enteritis. Enlarged MLN.	-	-	Infectious disease; salmonellosis + parasitic pneumonia
XT1352-15[H]	Nov 2015; East Sussex, England	Male Juvenile	Emaciated	250	Liver*, SI contents*, MLN*, throat, skin	Parasitic pneumonia.	-	40 x 15 x 5	*Refer to histopathological findings*
XT1416-15[H]	Dec 2014; Cambridgeshire, England	Male Subadult	Emaciated	180	Liver*, SI contents*, MLN*, throat, skin	Parasitic pneumonia. Enlarged MLN.	1.2	-	*Refer to histopathological findings*
XT1461-15	Dec 2015; Gloucestershire, England	Male Juvenile	Emaciated	222	Liver*, SI contents*, MLN*, throat, skin	Parasitic pneumonia.	-	40 x 10 x 10	Infectious disease; parasitic pneumonia<br><br>(*S*. Enteritidis PT11 unknown significance)

[C] Indicates cases in which co-infection with PT11 and PT66 has been confirmed or suspected.
[H] Indicates cases in which histopathological examination has been performed.
* Indicates tissues from which *Salmonella* Enteritidis was isolated.
SI: Small intestine, LI: Large intestine, LN: Lymph node, MLN Mesenteric lymph node

Supplementary Table S2: Summary of signalment and macroscopic abnormalities in hedgehogs with *Salmonella* Enteritidis PT66 infection

Case number	Date and location	Signalment	Body condition	Body weight (g)	Tissues sampled for microbiology *	Significant macroscopic abnormalities	MLN weight (g)	MLN dimensions (length x width x breath (mm))	Confirmed/ suspected cause of death
XT0035-14	Oct 2013; Perth & Kinross, Scotland	Female Adult	Fat	690	Liver, SI contents, MLN, skin*, LI contents, throat, pleural fluid, abdominal fluid, heart blood, inguinal LN	Abscessated MLN. Dermatophytosis of ventral skin caused by *Trichophyton terrestre*.	79.2	83 x - x -	Euthanasia due to Infectious disease; salmonellosis
XT0838-14	Oct 2012; Falkirk, Scotland	Female Adult	Fat	748	Liver*, SI contents, MLN*, skin, adrenal gland*	Severe periodontal disease. Enlarged MLN. Bilateral adrenal hyperplasia.	-	54 x 15 x 10	Euthanasia due to Infectious disease; salmonellosis
XT0839-14	Oct 2012; Stirling, Scotland	Male Adult	Thin	461	Liver, SI contents, MLN*	Abscessated MLN. Severe enteritis. Parasitic pneumonia. Moderate periodontal disease.	31.7	60 x 55 x-	Euthanasia due to Infectious disease; salmonellosis
XT0840-14	Aug 2012; Perth & Kinross, Scotland	Male Adult	Normal	634	Liver, SI contents, MLN*, skin, adrenal abscess*	Abscessated MLN x ≥ 2. Adrenal gland abscess.	-	39 x 21 x 18	Euthanasia due to Infectious disease; salmonellosis
XT0881-14[C,H]	Apr 2012; Fife, Scotland	Male Adult	Normal	585	Liver, SI contents, MLN*, skin, LI LN abscess*, urine	Abscessated MLN x ≥ 2. Acanthocephalans in peritoneal cavity.	114.1	70 x 48 x -	Euthanasia due to Infectious disease; salmonellosis

XT0882-14	Unknown 2011-2013; South Scotland	Male Adult	Normal	349	Liver*, SI contents*, MLN*, skin	Abscessated MLN x ≥ 2.	-	31 x 26 x 15	Euthanasia due to Infectious disease; salmonellosis
XT1113-14	Sep 2012; Fife, Scotland	Male Adult	Thin	616	Liver, SI contents, MLN*, skin, lung, gall bladder	Abscessated MLN x ≥ 2.	46.0	70 x 55 x -	Euthanasia due to Infectious disease; salmonellosis
XT1114-14	Aug 2012; Fife, Scotland	Male Adult	Normal	661	Liver, SI contents, MLN*, skin, axillary LN, lung	Abscessated MLN x ≥ 2. Granulomatous pneumonia. Axillary lymph node abscess.	22.0	65 x 30 x 18	Euthanasia due to Infectious disease; salmonellosis
XT1117-14	Jan 2013; West Lothian, Scotland	Male Adult	Thin	406	Liver, SI contents, MLN*, skin, mediastinal LN, lung	Abscessated MLN x ≥ 2. Abscessated mediastinal lymph nodes.	39.4	70 x 34 x 22	Euthanasia due to Infectious disease; salmonellosis
XT1118-14	Nov 2012; Angus, Scotland	Male Adult	Normal	649	Liver, SI contents, MLN*, skin	Abscessated MLN. Dermatitis.	30.2	53 x 42 x 22	Euthanasia due to Infectious disease; salmonellosis
XT1133-14	Aug 2012; Perth & Kinross, Scotland	Male Adult	Thin	489	Liver, SI contents*, MLN*, skin, mediastinal LN*	Abscessated MLN. Abscessated mediastinal lymph node. Intra-mural haemorrhage of small intestine serosa.	-	23 x 8 x 4	Euthanasia due to Infectious disease; salmonellosis
XT1136-14	Jul 2012; Midlothian, Scotland	Male Adult	Normal	671	Liver, SI contents, MLN*, skin, lung	Abscessated MLN. Heavy Ixodid sp. tick burden. Severe	-	79 x 67 x 34	Euthanasia due to Infectious disease;

						periodontal disease. Query adrenal hyperplasia?			salmonellosis
XT1139-14	May 2014; East Lothian, Scotland	Male Adult	Thin	558	Liver*, SI contents*, MLN*, skin, lung*	Abscessated MLN x ≥ 2. Granulomatous pneumonia. Severe periodontal disease.	23.1	44 x 39 x 27	Euthanasia due to Infectious disease; salmonellosis
XT1140-14	Sep 2013; Falkirk, Scotland	Male Adult	Thin	322	Liver*, SI contents*, MLN*, skin, heart blood*	Abscessated MLN x ≥ 2. Abscessated mediastinal lymph node.	-	20 x 20 x -	Euthanasia due to Infectious disease; salmonellosis
XT1141-14	Aug 2012; Angus, Scotland	Male Adult	Thin	626	Liver*, SI contents*, MLN*, skin, lung*	Abscessated MLN x ≥ 2.	-	37 x 18 x -	Euthanasia due to Infectious disease; salmonellosis
XT1142-14	Dec 2011; Fife, Scotland	Male Adult	Thin	450	Liver*, SI contents*, MLN*, skin, lung*, mediastinal LN*	Abscessated MLN x ≥ 2. Abscessated mediastinal lymph nodes. Tibia fracture. Mild periodontal disease.	38.5	60 x 39 x 24	Euthanasia due to Infectious disease; salmonellosis
XT1053-15[H]	Aug 2015; East Lothian, Scotland	Male Juvenile	Normal	481	Liver*, SI contents, MLN*, skin, lung, submandibular LN*, pleural fluid*, throat, spleen, heart blood	Abscessated MLN x ≥ 2. Submandibular lymph node abscess. High *Neotrombicula* sp. mite burden in ear canals.	18.5	61 x 47 x 14	*Euthanasia + refer to histopathological findings*
XT1148-15[H]	Sep 2015; West Lothian, Scotland	Female Adult	Normal	593	Liver*, SI contents*, MLN*, skin, LI contents*, throat	Abscessated MLN x ≥ 2. Gastritis (query gastric ulcers). Bilateral adrenal hyperplasia.	3.5	35 x 10 x 6	*Euthanasia + refer to histopathological findings*

XT1224-15[H]	Sep 2015; Stirling, Scotland	Female Juvenile	Thin	309	Liver*, SI contents, MLN*, skin, lung*, LI contents*, throat*, thymus*	Abscessated MLN x ≥ 2. Granulomatous pneumonia. Thymus abscess.	9.3	40 x 25 x 15	*Euthanasia + refer to histopathological findings*

[C] Indicates cases in which co-infection with PT11 and PT66 has been confirmed or suspected.
[H] Indicates cases in which histopathological examination has been performed.
* Indicates tissues from which *Salmonella* Enteritidis was isolated.
SI: Small intestine, LI: Large intestine, LN: Lymph node, MLN Mesenteric lymph node

Supplementary Table S3

Summary of sex, age and season of *Salmonella* Enteritidis PT11 and PT66 infections in hedgehogs from Great Britain, 2012-2015

Phage type	Sex	Age	Season
PT11	16 male, 11 female	16 juvenile, 3 subadult, 8 adult	spring 3, summer 8, autumn 13, winter 3
PT66	14 male, 4 female	2 juvenile, 16 adult	spring 1, summer 6, autumn 8, winter 2, unknown 1
Co-infection with PT11 & PT66	1 male	1 adult	spring 1

Supplementary Table S4: Microscopic abnormalities for cases with *Salmonella* Enteritidis PT11 infection

Case number	Significant microscopic abnormalities	Confirmed/ Suspected cause of death<br>Significance of *Salmonella* Enteritidis PT11 infection
**XT443-14**	Lungs: Bronchitis and bronchiolitis, proliferative, lymphoplasmacytic, multifocal, moderate with abundant intraluminal nematodes (morphology consistent with *Crenosoma* sp), and multifocal hypertrophy of the smooth muscle and alveolar oedema and haemorrhage<br><br>Trachea: Tracheitis, proliferative, lymphoplasmacytic and eosinophilic, severe with multifocal erosion, ulceration and hyperplasia of the epithelium and intralesional adult nematodes and intraluminal ova (morphology consistent with *Capillaria* spp.)<br><br>Nasal cavity, 1 section (slide 3): Rhinitis, multifocal to coalescing, lymphoplasmacytic, superficial, moderate<br><br>Mesenteric LN: Reactive lymphoid hyperplasia, diffuse, moderate<br><br>SI/LI: Enterocolitis, eosinophilic, multifocal, mild with intralesional metazoan parasites	Combination of poor body condition with moderate verminous pneumonia and severe verminous tracheitis<br><br>*S*. Enteritidis PT11 unknown significance
**XT1207-15**	Marked autolysis precluded meaningful interpretation of tissues.	Undetermined + emaciation<br><br>*S*. Enteritidis PT11 unknown significance
**XT1094-15**	Mesenteric LN: Reactive lymphoid hyperplasia, multifocal, moderate. Interpretation is hindered due to moderate degree of autolysis.<br><br>Retropharyngeal tissue: Myositis, steatitis and cellulitis, suppurative and lymphoplasmacytic, necrotizing,	Bacterial myositis, steatitis and cellulitis<br><br>Salmonellosis

	multifocal to coalescing, severe, chronic with myofiber degeneration, necrosis and loss, fibrosis and intralesional multinucleated giant cells and Gram negative bacilli bacterial colonies<br><br>Salmonella CSA-1 IHC: Retropharyngeal lymph node and surrounding tissue **positive**; brain, liver, spleen negative	
**XT995-15**	Urinary bladder: Cystitis, suppurative, diffuse, severe<br><br>Stomach: Mucosal erosion, ulceration and, congestion multifocal, moderate acute with few Gram negative and positive bacilli on the surface<br><br>Kidney: Nephritis, suppurative, multifocal to coalescing, severe	Bacterial nephritis and cystitis<br><br>Salmonellosis
**XT1270-15**	Lungs: Pneumonia, histiocytic, alveolar, focal, moderate and intrabronchial nematodes, multifocal, moderate (morphology consistent with *Crenosoma* sp).	Tissues too autolyzed to determine the cause of death.<br><br>*S*. Enteritidis PT11 unknown significance
**XT1315-15**	No significant abnormalities detected.	Death is due to severe trauma based on macroscopic findings (See Table 1)
**XT1352-15**	Lungs: Bronchial/bronchiolar nematodes, multifocal, severe (*Crenosoma* sp). Severe autolysis<br><br>Intraluminal nematodes in the SI	Death likely due to emaciation and verminous pneumonia<br><br>*S*. Enteritidis PT11 unknown significance
**XT1461-15**	Lungs: Pneumonia, verminous, multifocal, moderate, granulomatous with intralesional nematode larvae and intrabronchial, adult nematodes (morphology consistent with *Crenosoma* sp) with multifocal alveolar oedema and pulmonary congestion	Combination of poor body condition and verminous pneumonia<br><br>*S*. Enteritidis PT11 unknown significance

SI: Small intestine, LI: Large intestine, LN: Lymph node

Supplementary Table S5: Microscopic abnormalities for cases with *Salmonella* Enteritidis PT66 infection

Case number	Significant microscopic abnormalities	Confirmed/ Suspected cause of death<br>Significance of *Salmonella* Enteritidis infection
**XT1053-15**	Connective tissue attached to oesophagus: Cellulitis and steatitis, necrotizing, suppurative and lymphoplasmacytic, locally extensive, subacute with abundant intralesional Gram negative short bacilli and early fibrosis<br><br>Submandibular LN: Lymphadenitis, suppurative, with intralesional Gram negative bacilli<br><br>Mesenteric LN: Lymphadenitis, suppurative, necrotizing, multifocal, moderate with intralesional Gram negative bacilli<br><br>Salmonella CSA-1 IHC: Mesenteric LN and thymus and adrenal gland **positive**; bladder and brain negative	Euthanasia<br>Bacterial lymphadenitis<br><br>Salmonellosis
**XT1148-15**	Liver: Hepatitis, mononuclear (lymphohistiocytic), random multifocal, moderate with intralesional Gram negative bacilli<br><br>Intestine, suspected: Enterocolitis, transmural, necrotizing and suppurative, severe with intralesional mixed bacteria<br><br>Cervical LN: Lymphadenitis and cellulitis, locally extensive, severe with intralesional mixed bacterial<br><br>The degree of autolysis precluded the interpretation of the LI LN	Euthanasia<br>Bacterial lymphadenitis, hepatitis and enterocolitis<br><br>Salmonellosis
**XT1224-15**	Thymus: Thymitis, necrotizing, suppurative, diffuse, severe with intralesional Gram negative bacteria<br><br>Lung: Pneumonia and pleuritis, necrotizing, suppurative, locally extensive, severe with intralesional mixed bacteria and few adult nematodes and larvae<br><br>LI LN: Lymphadenitis, suppurative, multifocal with intralesional multinucleated giant cells and intralesional Gram negative bacilli	Euthanasia<br>Bacterial mesenteric and LI lymphadenitis, thymitis, pneumonia<br><br>Salmonellosis

	Mesenteric LN: Lymphadenitis, suppurative, severe, multifocal to coalescing, with intralesional Gram negative bacilli and abundant multinucleated giant cells<br><br>Salmonella CSA-1 IHC: Lung and thymus **positive**; brain negative	
**XT881-14**<br>(co-infection with S. Enteritidis PT66 and PT11)	Tissues too autolyze to assess	Euthanasia<br>Tissues to autolysed to determine the cause of death<br><br>*S*. Enteritidis PT11 and PT66 unknown significance

SI: Small intestine, LI: Large intestine, LN: Lymph node
